# 4-Meth­oxy­anilinium nitrate

**DOI:** 10.1107/S1600536811031862

**Published:** 2011-08-17

**Authors:** Hajer Rahmouni, Wajda Smirani Sta, S. Salem Al-Deyab, Mohamed Rzaigui

**Affiliations:** a Laboratoire de Chimie des Matériaux, Faculté des Sciences de Bizerte, 7021 Zarzouna Bizerte, Tunisia; b Petrochemical Research Chair, College of Science, King Saud University, Riyadh, Saudi Arabia

## Abstract

The title compound, C_7_H_10_NO^+^·NO_3_^−^, crystallized with two *p*-ansidinium cations and two nitrate anions in the asymmetric unit. As well as Columbic and van der Waals forces, moleucles inter­act *via* multiple bifurcated N—H⋯O hydrogen bonds that help consolidate the crystal packing, resulting in a three-dimensional network.

## Related literature

For background to anisidine, see: Li *et al.* (2001 ▶). For applications of nitrates, see: Kapoor *et al.* (2008 ▶). Association of both entities could lead to new molecular salts with inter­esting physical and chemical properties, see: Wilkes *et al.* (1985 ▶). For related structures, see: Ben Amor *et al.* (1995 ▶); Liu *et al.* (2011 ▶).
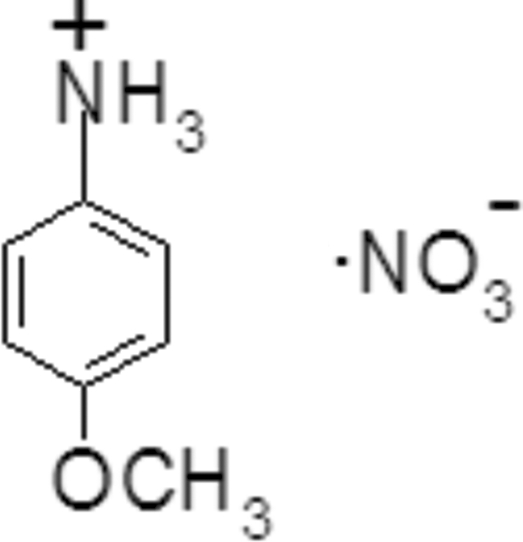


## Experimental

### 

#### Crystal data

C_7_H_10_NO^+^·NO_3_^−^
*M* *_r_* = 186.17
Monoclinic, 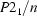

*a* = 14.724 (2) Å
*b* = 7.304 (3) Å
*c* = 17.509 (2) Å
β = 112.84 (2)°
*V* = 1735.3 (8) Å^3^
*Z* = 8
Ag *K*α radiation
λ = 0.56085 Å
μ = 0.07 mm^−1^
*T* = 293 K
0.35 × 0.25 × 0.20 mm

#### Data collection

Enraf–Nonius TurboCAD-4 diffractometer
12462 measured reflections
8244 independent reflections
2756 reflections with *I* > 2σ(*I*)
*R*_int_ = 0.033
2 standard reflections every 120 min  intensity decay: 5%

#### Refinement

*R*[*F* ^2^ > 2σ(*F* ^2^)] = 0.069
*wR*(*F* ^2^) = 0.219
*S* = 0.93
8244 reflections
235 parameters
H-atom parameters not refined
Δρ_max_ = 0.53 e Å^−3^
Δρ_min_ = −0.25 e Å^−3^

### 

Data collection: *CAD-4 EXPRESS* (Enraf–Nonius, 1994 ▶); cell refinement: *CAD-4 EXPRESS*; data reduction: *XCAD4* (Harms & Wocadlo, 1996 ▶); program(s) used to solve structure: *SHELXS97* (Sheldrick, 2008 ▶); program(s) used to refine structure: *SHELXL97* (Sheldrick, 2008 ▶); molecular graphics: *ORTEPIII* (Burnett & Johnson, 1996 ▶) and *ORTEP-3 for Windows* (Farrugia, 1997 ▶); software used to prepare material for publication: *WinGX* (Farrugia, 1999 ▶).

## Supplementary Material

Crystal structure: contains datablock(s) I, global. DOI: 10.1107/S1600536811031862/fl2352sup1.cif

Structure factors: contains datablock(s) I. DOI: 10.1107/S1600536811031862/fl2352Isup2.hkl

Supplementary material file. DOI: 10.1107/S1600536811031862/fl2352Isup3.cml

Additional supplementary materials:  crystallographic information; 3D view; checkCIF report

## Figures and Tables

**Table 1:** Hydrogen-bond geometry (Å, °)

*D*—H⋯*A*	*D*—H	H⋯*A*	*D*⋯*A*	*D*—H⋯*A*
N1—H1*A*⋯O4^i^	0.89	2.25	2.823 (3)	122
N1—H1*A*⋯O7^ii^	0.89	2.52	2.979 (3)	113
N1—H1*B*⋯O6^iii^	0.89	2.26	2.843 (3)	123
N1—H1*B*⋯O5^iv^	0.89	2.12	2.903 (3)	146
N1—H1*C*⋯O7^v^	0.89	2.14	2.935 (3)	148
N2—H2*A*⋯O3^i^	0.89	2.08	2.967 (3)	177
N2—H2*A*⋯O4^i^	0.89	2.55	3.187 (3)	129
N2—H2*B*⋯O6^vi^	0.89	2.46	3.070 (3)	127
N2—H2*B*⋯O7^vi^	0.89	2.22	3.083 (3)	163
N2—H2*C*⋯O3	0.89	2.07	2.891 (2)	152
C9—H9⋯O8^v^	0.93	2.48	3.223 (3)	137

Symmetry codes: (i) 
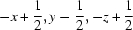
; (ii) 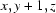; (iii) 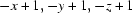; (iv) 
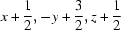
; (v) 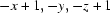; (vi) 
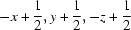
.

**Fig. 1.**
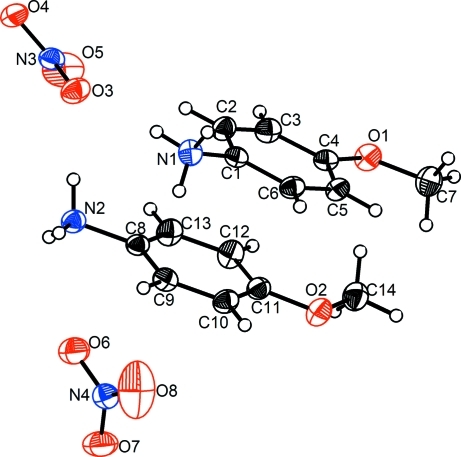
The assymetric unit of the title compound, with the atom numbering scheme. Displacement ellipsoids are drawn at the 30% probability level. H atoms are represented as small sphere of arbitrary radii.

**Fig. 2.**
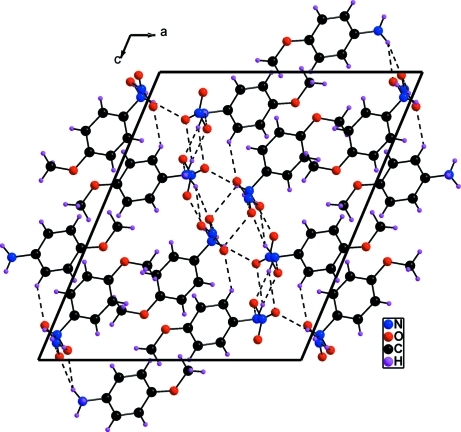
Projection of the crystal packing along the b axis.

## supplementary crystallographic information

### Comment

Anisidine is used in various areas such as the production of polymers of high solubility which are interesting materials in electroconductivity and thermostability (Li *et al.*, 2001). Nitrates also have many applications such as explosives and  pyrotechnics and they can be powerful oxidizers (Kapoor *et al.*, 2008).  Association of both entities could lead to novel hybrid compounds with interesting physical and chemical properties (Wilkes *et al.*, 1985). The exploitation of these materials requires knowledge of not only their electronic properties but also of their atomic arrangements.

In this paper, we report crystal structure of the interaction product of [*p*-ANI] and nitric acid (I).  As shown in Fig.1, the asymmetric unit of (I) contains two nitrate anions and two [*p*-ANIH]^+^ cations interconnected by N—H···O and C—H···O hydrogen bonds (Table 1)). Geometrical characteristics of the two independent nitrate anions are slightly different. In one the N—O distances (N_3_/O_3_/O_4_/O_5_) range from 1.202 (3) to 1.268 (2) Å while in the other one the N—O distances (N_4_/O_6_/O_7_/O_8_) range from 1.177 (3) to 1.268 (2) Å.  Examination of the [*p*-ANIH]^+^ cations shows that the bond distances and angles show no significant difference from those obtained in other structures involving the same organic groups (Ben Amor *et al.*, 1995). The phenyl rings of these cations are planar with a maximum atomic deviation of ±0.00027 Å and a dihedral angle between them of 8.17°.

The crystal packing shows how each nitrate anion is connected to three [*p*-ANIH]^+^ cations by N—H···O hydrogen bonding interactions (Table 1, Fig.2). It is noteworthy that the oxygen atom of the shortest bond (N_4_—O_8_: 1.177 (3) Å) does not participate to the hydrogen bonding network. A similar situation was observed in C_36_H_40_N_5_O_6_NO_3_.2C_2_H_5_OH (Liu *et al.*, 2011), where the N—O bond length is 1.186 (8) Å.

### Experimental

An ethanolic solution of *p*-anisidine [*p*-ANI] (10 mmol, in 10 ml) was added drop wise to a magnetically stirred aqueous solution of nitric acid HNO_3_ (10 mmol, 20 ml). The resultant solution was then slowly evaporated at room temperature (295 K).  After a  few days, colorless crystals ofn(I) appeared that were suitable for *X*– ray diffraction measurements.

### Refinement

All H atoms were positioned geometrically and treated as riding on their parent atoms, [N–H = 0.89, C–H =0.96 Å (CH_3_ ) with with *U*_iso_(H) = 1.5Ueq and C–H =0.96 Å (Ar–H), with *U*_iso_(H) = 1.5Ueq],.

### Crystal data

**Table:**

C_7_H_10_NO^+^·NO_3_^−^	*F*(000) = 784
*M**_r_* = 186.17	*D*_x_ = 1.425 Mg m^−^^3^
Monoclinic, *P*2_1_/*n*	Ag *K*α radiation, λ = 0.56085 Å
Hall symbol: -P 2yn	Cell parameters from 25 reflections
*a* = 14.724 (2) Å	θ = 9–11°
*b* = 7.304 (3) Å	µ = 0.07 mm^−^^1^
*c* = 17.509 (2) Å	*T* = 293 K
β = 112.84 (2)°	Block, brown
*V* = 1735.3 (8) Å^3^	0.35 × 0.25 × 0.20 mm
*Z* = 8	

### Data collection

**Table:**

Enraf–Nonius TurboCAD-4 diffractometer	*R*_int_ = 0.033
Radiation source: fine-focus sealed tube	θ_max_ = 28.0°, θ_min_ = 2.4°
graphite	*h* = −24→23
non–profiled ω scans	*k* = −3→12
12462 measured reflections	*l* = −2→29
8244 independent reflections	2 standard reflections every 120 min
2756 reflections with *I* > 2σ(*I*)	intensity decay: 5%

### Refinement

**Table:**

Refinement on *F*^2^	Primary atom site location: structure-invariant direct methods
Least-squares matrix: full	Secondary atom site location: difference Fourier map
*R*[*F*^2^ > 2σ(*F*^2^)] = 0.069	Hydrogen site location: inferred from neighbouring sites
*wR*(*F*^2^) = 0.219	H-atom parameters not refined
*S* = 0.93	*w* = 1/[σ^2^(*F*_o_^2^) + (0.0981*P*)^2^] where *P* = (*F*_o_^2^ + 2*F*_c_^2^)/3
8244 reflections	(Δ/σ)_max_ < 0.001
235 parameters	Δρ_max_ = 0.53 e Å^−^^3^
0 restraints	Δρ_min_ = −0.25 e Å^−^^3^

### Special details

**Table:**

Geometry. All esds (except the esd in the dihedral angle between two l.s. planes) are estimated using the full covariance matrix. The cell esds are taken into account individually in the estimation of esds in distances, angles and torsion angles; correlations between esds in cell parameters are only used when they are defined by crystal symmetry. An approximate (isotropic) treatment of cell esds is used for estimating esds involving l.s. planes.
Refinement. Refinement of *F*^2^ against ALL reflections. The weighted *R*-factor *wR* and goodness of fit *S* are based on *F*^2^, conventional *R*-factors *R* are based on *F*, with *F* set to zero for negative *F*^2^. The threshold expression of *F*^2^ > σ(*F*^2^) is used only for calculating *R*-factors(gt) *etc*. and is not relevant to the choice of reflections for refinement. *R*-factors based on *F*^2^ are statistically about twice as large as those based on *F*, and *R*- factors based on ALL data will be even larger.

### Fractional atomic coordinates and isotropic or equivalent isotropic displacement parameters (Å^2^)

**Table:**

	*x*	*y*	*z*	*U*_iso_*/*U*_eq_	
N4	0.45826 (13)	−0.1031 (3)	0.55952 (10)	0.0482 (4)	
O7	0.42497 (16)	−0.2382 (2)	0.51123 (13)	0.0797 (6)	
O8	0.53055 (17)	−0.0919 (6)	0.61993 (13)	0.1391 (13)	
C8	0.32040 (12)	0.3581 (2)	0.13224 (11)	0.0384 (4)	
N2	0.23464 (11)	0.4351 (2)	0.14327 (10)	0.0439 (4)	
H2A	0.2350	0.4003	0.1921	0.066*	
H2B	0.1798	0.3952	0.1030	0.066*	
H2C	0.2369	0.5567	0.1414	0.066*	
O2	0.56519 (10)	0.1375 (2)	0.10671 (9)	0.0554 (4)	
C9	0.38580 (13)	0.2496 (3)	0.19357 (12)	0.0425 (4)	
H9	0.3755	0.2252	0.2418	0.051*	
C10	0.46634 (14)	0.1778 (3)	0.18263 (12)	0.0460 (4)	
H10	0.5105	0.1038	0.2235	0.055*	
C11	0.48208 (13)	0.2150 (3)	0.11122 (11)	0.0411 (4)	
C12	0.41615 (15)	0.3222 (3)	0.04975 (12)	0.0518 (5)	
H12	0.4260	0.3461	0.0014	0.062*	
C13	0.33478 (15)	0.3940 (3)	0.06103 (12)	0.0503 (5)	
H13	0.2900	0.4667	0.0200	0.060*	
C14	0.58718 (17)	0.1818 (4)	0.03694 (15)	0.0594 (6)	
H14A	0.6462	0.1195	0.0409	0.089*	
H14B	0.5966	0.3116	0.0354	0.089*	
H14C	0.5336	0.1444	−0.0127	0.089*	
O1	0.67037 (11)	0.5777 (2)	0.15815 (8)	0.0509 (4)	
C1	0.56621 (13)	0.5870 (2)	0.34540 (11)	0.0381 (4)	
C4	0.63880 (13)	0.5719 (3)	0.22190 (11)	0.0396 (4)	
C5	0.68716 (14)	0.4806 (3)	0.29573 (12)	0.0422 (4)	
H5	0.7438	0.4135	0.3038	0.051*	
N1	0.53081 (12)	0.6024 (3)	0.41235 (11)	0.0482 (4)	
H1A	0.4758	0.6687	0.3952	0.072*	
H1B	0.5766	0.6567	0.4558	0.072*	
H1C	0.5185	0.4912	0.4268	0.072*	
C2	0.51623 (14)	0.6757 (3)	0.27082 (12)	0.0459 (5)	
H2	0.4588	0.7405	0.2625	0.055*	
C6	0.65044 (14)	0.4896 (3)	0.35843 (12)	0.0426 (4)	
H6	0.6831	0.4298	0.4086	0.051*	
C3	0.55199 (14)	0.6674 (3)	0.20970 (12)	0.0467 (5)	
H3	0.5182	0.7258	0.1594	0.056*	
C7	0.76321 (18)	0.4981 (4)	0.17243 (15)	0.0588 (6)	
H7A	0.7774	0.5105	0.1236	0.088*	
H7B	0.7619	0.3706	0.1854	0.088*	
H7C	0.8133	0.5591	0.2180	0.088*	
N3	0.21447 (12)	0.9114 (2)	0.14231 (11)	0.0462 (4)	
O3	0.27158 (11)	0.8115 (2)	0.19742 (10)	0.0577 (4)	
O4	0.17418 (11)	1.0419 (2)	0.16528 (12)	0.0642 (5)	
O5	0.19753 (18)	0.8822 (3)	0.07051 (12)	0.0925 (7)	
O6	0.40423 (13)	0.0358 (3)	0.53394 (14)	0.0787 (5)	

### Atomic displacement parameters (Å^2^)

**Table:**

	*U*^11^	*U*^22^	*U*^33^	*U*^12^	*U*^13^	*U*^23^
N4	0.0599 (10)	0.0482 (10)	0.0424 (9)	0.0005 (9)	0.0262 (8)	0.0083 (8)
O7	0.1130 (15)	0.0479 (10)	0.0942 (14)	−0.0030 (10)	0.0575 (12)	−0.0063 (10)
O8	0.0827 (14)	0.259 (4)	0.0518 (11)	0.0282 (18)	0.0007 (11)	0.0389 (16)
C8	0.0372 (8)	0.0337 (9)	0.0392 (9)	−0.0009 (7)	0.0092 (7)	−0.0012 (7)
N2	0.0417 (8)	0.0402 (9)	0.0448 (8)	0.0024 (7)	0.0112 (7)	0.0001 (7)
O2	0.0528 (8)	0.0581 (10)	0.0563 (9)	0.0137 (7)	0.0223 (7)	0.0056 (7)
C9	0.0422 (9)	0.0421 (10)	0.0370 (9)	−0.0007 (8)	0.0085 (7)	0.0054 (8)
C10	0.0465 (10)	0.0422 (10)	0.0438 (10)	0.0081 (8)	0.0116 (8)	0.0084 (9)
C11	0.0403 (9)	0.0370 (10)	0.0427 (9)	0.0024 (7)	0.0124 (8)	0.0001 (8)
C12	0.0546 (11)	0.0610 (13)	0.0405 (10)	0.0101 (10)	0.0191 (9)	0.0117 (10)
C13	0.0478 (10)	0.0547 (12)	0.0419 (10)	0.0105 (9)	0.0104 (8)	0.0142 (9)
C14	0.0585 (12)	0.0624 (14)	0.0642 (13)	0.0047 (11)	0.0312 (11)	−0.0008 (12)
O1	0.0584 (8)	0.0552 (9)	0.0418 (7)	0.0062 (7)	0.0221 (6)	0.0036 (6)
C1	0.0399 (8)	0.0334 (9)	0.0393 (8)	−0.0047 (7)	0.0136 (7)	−0.0037 (7)
C4	0.0445 (9)	0.0329 (9)	0.0389 (9)	−0.0022 (7)	0.0134 (7)	−0.0021 (7)
C5	0.0430 (9)	0.0385 (10)	0.0444 (10)	0.0074 (8)	0.0161 (8)	0.0035 (8)
N1	0.0446 (8)	0.0523 (10)	0.0484 (9)	−0.0010 (7)	0.0189 (7)	−0.0013 (8)
C2	0.0388 (9)	0.0430 (10)	0.0501 (10)	0.0058 (8)	0.0107 (8)	0.0016 (9)
C6	0.0457 (9)	0.0372 (9)	0.0411 (9)	0.0049 (8)	0.0125 (8)	0.0055 (8)
C3	0.0475 (10)	0.0457 (11)	0.0400 (9)	0.0037 (9)	0.0095 (8)	0.0046 (8)
C7	0.0598 (12)	0.0687 (15)	0.0520 (12)	0.0045 (11)	0.0261 (10)	−0.0026 (11)
N3	0.0418 (8)	0.0362 (8)	0.0506 (9)	−0.0026 (7)	0.0070 (7)	0.0007 (7)
O3	0.0564 (8)	0.0490 (8)	0.0568 (9)	0.0122 (7)	0.0100 (7)	0.0069 (7)
O4	0.0537 (8)	0.0401 (8)	0.0936 (12)	0.0076 (7)	0.0230 (8)	−0.0016 (8)
O5	0.1142 (16)	0.0904 (15)	0.0516 (10)	−0.0027 (13)	0.0089 (10)	−0.0085 (10)
O6	0.0653 (10)	0.0539 (10)	0.1067 (14)	0.0002 (8)	0.0223 (10)	−0.0067 (10)

### Geometric parameters (Å, °)

**Table:**

N4—O8	1.177 (3)	O1—C4	1.366 (2)
N4—O6	1.259 (3)	O1—C7	1.416 (3)
N4—O7	1.268 (2)	C1—C6	1.370 (3)
C8—C13	1.369 (3)	C1—C2	1.386 (3)
C8—C9	1.381 (2)	C1—N1	1.460 (3)
C8—N2	1.462 (2)	C4—C5	1.381 (3)
N2—H2A	0.8900	C4—C3	1.398 (3)
N2—H2B	0.8900	C5—C6	1.401 (3)
N2—H2C	0.8900	C5—H5	0.9300
O2—C11	1.378 (2)	N1—H1A	0.8900
O2—C14	1.417 (3)	N1—H1B	0.8900
C9—C10	1.376 (3)	N1—H1C	0.8900
C9—H9	0.9300	C2—C3	1.365 (3)
C10—C11	1.384 (3)	C2—H2	0.9300
C10—H10	0.9300	C6—H6	0.9300
C11—C12	1.379 (3)	C3—H3	0.9300
C12—C13	1.390 (3)	C7—H7A	0.9600
C12—H12	0.9300	C7—H7B	0.9600
C13—H13	0.9300	C7—H7C	0.9600
C14—H14A	0.9600	N3—O5	1.202 (3)
C14—H14B	0.9600	N3—O3	1.241 (2)
C14—H14C	0.9600	N3—O4	1.268 (2)
			
O8—N4—O6	119.3 (3)	C4—O1—C7	117.01 (16)
O8—N4—O7	129.4 (3)	C6—C1—C2	121.00 (18)
O6—N4—O7	111.25 (18)	C6—C1—N1	119.25 (16)
C13—C8—C9	120.71 (18)	C2—C1—N1	119.72 (17)
C13—C8—N2	119.57 (16)	O1—C4—C5	124.37 (17)
C9—C8—N2	119.72 (17)	O1—C4—C3	116.13 (17)
C8—N2—H2A	109.5	C5—C4—C3	119.50 (18)
C8—N2—H2B	109.5	C4—C5—C6	119.69 (17)
H2A—N2—H2B	109.5	C4—C5—H5	120.2
C8—N2—H2C	109.5	C6—C5—H5	120.2
H2A—N2—H2C	109.5	C1—N1—H1A	109.5
H2B—N2—H2C	109.5	C1—N1—H1B	109.5
C11—O2—C14	117.42 (16)	H1A—N1—H1B	109.5
C10—C9—C8	119.27 (18)	C1—N1—H1C	109.5
C10—C9—H9	120.4	H1A—N1—H1C	109.5
C8—C9—H9	120.4	H1B—N1—H1C	109.5
C9—C10—C11	120.42 (17)	C3—C2—C1	119.49 (17)
C9—C10—H10	119.8	C3—C2—H2	120.3
C11—C10—H10	119.8	C1—C2—H2	120.3
O2—C11—C12	124.06 (18)	C1—C6—C5	119.58 (17)
O2—C11—C10	115.76 (17)	C1—C6—H6	120.2
C12—C11—C10	120.17 (18)	C5—C6—H6	120.2
C11—C12—C13	119.17 (18)	C2—C3—C4	120.71 (18)
C11—C12—H12	120.4	C2—C3—H3	119.6
C13—C12—H12	120.4	C4—C3—H3	119.6
C8—C13—C12	120.25 (18)	O1—C7—H7A	109.5
C8—C13—H13	119.9	O1—C7—H7B	109.5
C12—C13—H13	119.9	H7A—C7—H7B	109.5
O2—C14—H14A	109.5	O1—C7—H7C	109.5
O2—C14—H14B	109.5	H7A—C7—H7C	109.5
H14A—C14—H14B	109.5	H7B—C7—H7C	109.5
O2—C14—H14C	109.5	O5—N3—O3	120.8 (2)
H14A—C14—H14C	109.5	O5—N3—O4	122.17 (19)
H14B—C14—H14C	109.5	O3—N3—O4	117.03 (18)

### Hydrogen-bond geometry (Å, °)

**Table:**

*D*—H···*A*	*D*—H	H···*A*	*D*···*A*	*D*—H···*A*
N1—H1A···O4^i^	0.89	2.25	2.823 (3)	122
N1—H1A···O7^ii^	0.89	2.52	2.979 (3)	113
N1—H1B···O6^iii^	0.89	2.26	2.843 (3)	123
N1—H1B···O5^iv^	0.89	2.12	2.903 (3)	146
N1—H1C···O7^v^	0.89	2.14	2.935 (3)	148
N2—H2A···O3^i^	0.89	2.08	2.967 (3)	177
N2—H2A···O4^i^	0.89	2.55	3.187 (3)	129
N2—H2B···O6^vi^	0.89	2.46	3.070 (3)	127
N2—H2B···O7^vi^	0.89	2.22	3.083 (3)	163
N2—H2C···O3	0.89	2.07	2.891 (2)	152
C9—H9···O8^v^	0.93	2.48	3.223 (3)	137

Symmetry codes: (i) −*x*+1/2, *y*−1/2, −*z*+1/2; (ii) *x*, *y*+1, *z*; (iii) −*x*+1, −*y*+1, −*z*+1; (iv) *x*+1/2, −*y*+3/2, *z*+1/2; (v) −*x*+1, −*y*, −*z*+1; (vi) −*x*+1/2, *y*+1/2, −*z*+1/2.
